# Quantitative Immunoexpression of EGFR in Oral Potentially Malignant Disorders: Oral Leukoplakia and Oral Submucous Fibrosis

**DOI:** 10.15171/joddd.2015.031

**Published:** 2015-09-16

**Authors:** Naga Jyothi Meka, Sridevi Ugrappa, Nagalaxmi Velpula, Sravan Kumar, Kotya Naik Maloth, Srikanth Kodangal, Lalitha ch, Stuti Goyal

**Affiliations:** ^1^Senior lecturer, Department of Oral Medicine and Radiology, Aditya dental college, India; ^2^Lecturer, faculty of dentistry, Aimst Dental Institute, Aimst University, Semeling, 08100, Bedong, Malaysia; ^3^Professor and Head of the Department, Department of Oral Medicine and Radiology, Sri Sai College of Dental Surgery, India; ^4^Assistant Professor, Department of Oral and Maxillofacial Pathology, Sri Sai College of Dental Surgery, India; ^5^Assistant Professor, Department of Oral Medicine and Radiology, Mamata Dental College, India; ^6^Associate Professor, Department of Oral Medicine and Radiology, Sri Sai College of Dental Surgery, India; ^7^Assistant professor, department of oral medicine and radiology, Sri Sai College of dental surgery, India

**Keywords:** Epidermal growth factor receptor, neoplastic process, oral leukoplakia, oral submucous fibrosis

## Abstract

***Background and aims.*** Many oral squamous cell carcinomas develop from potentially malignant disorders (PMDs)which include a variety of lesions and conditions characterized by an increased risk for malignant transformation. Thisstudy evaluated the quantitative expression of EGFR in normal oral mucosa, oral leukoplakia and oral submucous fibrosis to predict the malignant risk in compliance with the intensity of staining with EGFR.

***Materials and methods.*** Thirty subjects were included in the study, consisting of 10 oral leukoplakia (OL), 10 oral submucous fibrosis (OSMF) and 10 normal oral mucosa (NOM) as the control group. Owing to the histopathological confirmation of precancerous state of tissue, 4-μm-thick sections of tissue were taken from paraffin-embedded wax blocks for immunohistochemical staining for EGFR.

***Results.*** All the control cases showed positive expression for EGFR, while 20% of oral leukoplakia and 40% of OSMF cases showed strong expression (3+), 40% of OL and 30% of OSMF cases showed weak expression (2+), and 40% of OLand 30% of OSMF cases showed poor expression (1+) compared to controls (P=0.012).

***Conclusion.*** EGFR expression levels in the premalignant lesion appear to be a sensitive factor in predicting the neoplastic potential. This suggests that EGFR may serve as a biological marker to identify high-risk subgroups and guide prophylactic therapy with chemopreventive drugs or surgical intervention to prevent progression to carcinoma. Hence, further investigations in the direction of chemopreventive trials with a larger sample size are suggested to determine its role in the head and neck tumorigenesis.

## Introduction

With an increase in the abuse of various oral habitual products in India over the past few decades, the incidence of oral potentially malignant disorder rates, including those of leukoplakia, oral submucous fibrosis and squamous cell carcinoma, have increased. The following disorders are regarded as being potentially malignant: 1) leukoplakia/erythroplakia, 2) submucous fibrosis, 3) palatal lesions in reverse smokers, and although still somewhat questionable 4) lichen planus, and 5) discoid lupus erythematosus.^1^Oral carcinoma which is a leading cause of death due to cancer worldwide^2^ is usually preceded by the occurrence of various potentially malignant disorders (PMDs) with variable morbidity and mortality rates. Beyond prevention, early detection is the most crucial determinant for the successful treatment, better prognosis and survival of cancer. Yet current methodologies for cancer diagnosis based upon pathological examination alone are insufficient for detecting early tumor progression and molecular transformation.^2^

Most common among them include oral leukoplakia (OL) and oral submucous fibrosis (OSMF). Malignant transformation of OL is 1-20% over 1-30 years, while that of OSMF is 7-13%.^3^

OL can be defined as a white patch or plaque of questionable risk that cannot be characterized clinically or pathologically as any other known disease.^4^ Upon biopsy, some OLs may exhibit epithelial dysplasia that is likely associated with progression to cancer. OSMF is an insidious chronic inflammatory disease of the oral mucosa and can be diagnosed clinically based on the presence of vertical palpable fibrous bands in the oral mucosa, depapillation of the tongue and restricted movements.^3^

Many molecular studies have explored a number of genes that are altered, amplified, deregulated in the expression or deleted in the head and neck tumorigenesis and several hypotheses have been put forward to identify specific prognostic markers predicting the malignant potential of a more widely prevalent oral PMDs^5^ along with the early identification of second primary tumors that develop from clonal expansion explained by the hypothesis of field cancerization.

Some of the PMDs occurring in the oral cavity show malignant transformation while some do not progress to malignancy, which can be explained by the individual variations in the genetic susceptibility and other immune-pathogenic mechanisms in the body. However, the need for the specific molecular biomarkers at the cellular and genetic level targeting the dysplastic oral epithelium has increased consequent to the increased prevalence of habit-induced oral cancer. Among many biological markers, EGFR was most studied due to its unique role in the oral carcinogenesis as it incites all the cellular events like uncoordinated cellular proliferation, angiogenesis, invasion, metastasis, increased cell survival and evasion of apoptosis.^6^

The epidermal growth factor receptor (EGFR) belongs to the ErbB family of receptor tyrosine kinases. The EGFR gene can be mapped to chromosome 7p11.2 and encodes a 170-kDa transmembrane glycoprotein. Alterations in the function of EGFR have been linked with oncogenic transformation, autonomous cell growth, invasion, angiogenesis and development of metastases in several cancers and are key characteristics of tumors.^5^ Malignant oral keratinocytes possess 5 to 50 times more EGFR than their normal counterparts.^3^ It is found at abnormally high levels on the surface of many types of cancer cells, so these cells may divide excessively in the presence of epidermal growth factor. It is also termed as ErbB1 and HER1.^6^

In recent years, EGFR has been considered a promising target for monoclonal antibody therapy. High EGFR expression has been correlated with tumor size, metastasis and survival.^7^Oral squamous cell carcinoma in the head and neck region over-expressing EGFR exhibits a higher proportion of complete responses to chemotherapy than other malignancies with low-level EGFR expression.^8^ Over-expression of EGFR presumably due to higher intrinsic proliferative activity could result in higher sensitivity to drug therapy cytotoxic to cells undergoing mitogenesis.^7^

Considering its major role in carcinogenesis, this study investigated the clinical significance of expression of EGFR in habit-associated (chewing and smoking form of tobacco) oral PMDs. The aim of the present study was to evaluate the expression of EGFR in habit induced oral leukoplakia and oral submucous fibrosis patients.

## Materials and Methods

### Selection of Samples 

The present cross-sectional study included patients visiting the Department of Oral Medicine and Radiology. A total of 30 samples were included in the study and divided into 3 groups: group I consisted of 10 OL, group II consisted of 10 OSMF and group III consisted of 10 normal oral mucosa (NOM) as a control group. Demographic data, personal habit history of tobacco smoking and chewing, betel-nut chewing and use of pan masala and alcohol consumption as well as clinical appearance of the lesion was recorded in the scheduled interview after acquiring the ethical clearance from our institution.

After evaluating the patient clinically and obtaining informed consent, an incisional biopsy was performed for OL and OSMF (PMDs). The histological diagnosis of the cases was carried out based on the latest WHO consensus criteria based on architectural and cytologic changes in the epithelium.^9^

### Inclusion Criteria

1. All cases of clinically proven homogenous leukoplakia involving buccal mucosa were included in group I. Histologically, presence of dysplasia in samples of OL was considered for the study.
2. All cases of clinically proven blanching involving most of the sites of oral cavity were included in group II. Histologically, epithelial atrophy, keratosis and dysplasia in samples of OSMF were considered for the study.
3. In control group III tissue samples were collected from gingival tissues of premolars extracted for orthodontic purposes.

### Exclusion Criteria 

All the cases associated with tobacco consumption habit, clinically showing benign hyperkeratosis with no histological dysplasia, were excluded from the study.

Subsequent to the histopathological confirmation of precancerous or preneoplastic state of the tissue, 4-µm thick sections of tissue were taken from paraffin-embedded wax blocks onto poly-L-lysine adhesive coated slides with the help of soft tissue microtome and incubated for 3 hours at 50-60°C in a slide warmer for proper adhesion of the section. Microwave antigen retrieval was carried out on sections in 0.01 mol/L sodium citrate buffer (pH=6.0), as per standard protocol, and endogenous peroxides were blocked using 3% hydrogen peroxide for 30 minutes. Immunohistochemical staining for EGFR was carried out by streptavidin-biotin method with appropriate positive, negative, and reagent controls. The tissue sections were kept at 37°C and fixed overnight at 600°C before immunohistochemistry. Dewaxing was carried out in xylene and rehydration was carried out in gradient alcohol (absolute alcohol of 70% and 50%) and finally in distilled water for 5 minutes each. Blocking was carried out by using 3% H_2_O_2_ in methanol for 30 minutes. Antigen retrieval was carried out using citrate buffer (pH=6.0) method to optimize staining for 120 minutes at 98°C. The sections were immunostained with primary polyclonal antibody for EGFR (Biogenex, Bangalore, India). Sections were incubated overnight at 4°C with primary antibody in a humid chamber. The following day, the sections were stained using labeled streptavidin-biotin biogenex kit (DAKO LSAB + system, K0679) with modified timings, and the sections were incubated for 2 hours in the corresponding biotinylated secondary antibody solution, followed by conjugated streptavidin horseradish peroxidase complex for 1 hour. Bound peroxidase was revealed using 0.05% 3-diaminobenzedinetetrahydro (DAB) in TBS. The sections were dehydrated, cleared and mounted.

Assessment of immune reaction of EGFR staining was performed using double-headed light microscope at ×40. The criteria used to define EGFR antigen-positive cells were brown staining in dysplastic cell membranes. Presence of immunostaining in the cell membrane of various layers of epithelium was evaluated in randomized 6 fields/intensity of positively stained cells as percentage expression at ×40 and graded as 0 (under 10% positively stained cells), 1+ (10–25% positively stained cells: weak expression), 2+ (25–50% positively stained cells: mild to moderate expression), 3+ (50–75% positive cells: moderate to strong expression).

Interpretation of EGFR immunoreactivity was carried out by two independent observers and the results were subjected to statistical analysis. The inter-examiner reliability in recording expression of EGFR was found to be 0.96 (intra-class correlation co-efficient), implying good agreement between the two observers. Hence, the study was proceeded with a single examiner. All the calculations were performed using IBM SPSS statistical software package (SPSS 14.0 for Windows, SPSS Inc, Chicago, IL, USA). Data were analyzed by Pearson and chi-squared tests. Statistical significance was defined at P<0.05.

## Results

The study group comprised 20 tissue samples of OL, OSMF and 10 samples of normal oral mucosa as controls. The criteria used to define EGFR staining in the cell membrane of various layers of epithelium and the intensity of staining were considered in the immunohistochemical evaluation, irrespective of the grade of dysplasia. The data obtained from the study were compiled, tabulated and subjected to statistical analysis. The results are presented in the following manner:

### Distribution of Study Subjects Based on Age

**Figure 1.**
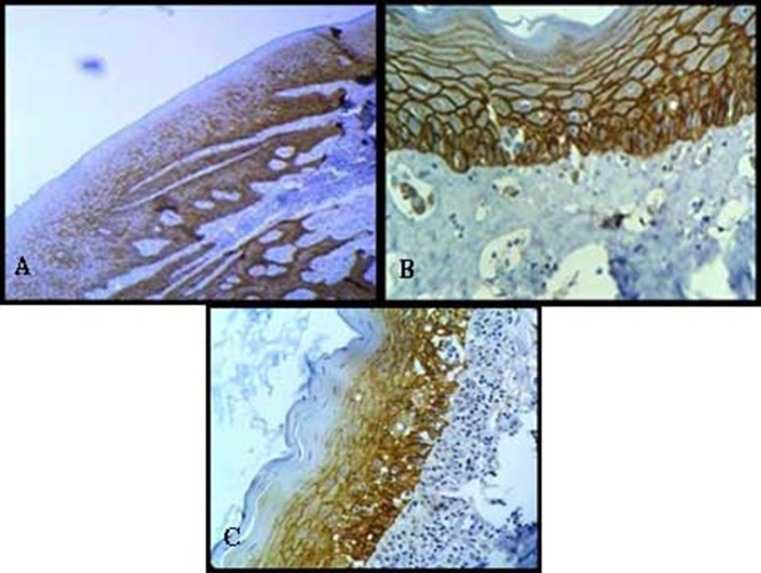
Age distribution in groups. The study group included 20 tissue samples of OL and OSMF. The mean age of OL was 41.1 years and that of OSMF was 30.9 years. The control group included 10 subjects with a mean age of 37.3 years.

The study group included 20 tissue samples of OL and OSMF. Among those with leukoplakia, 10 study subjects in the 20-70-year age range, with a mean age of 41.10 years. In the OSMF, 10 subjects in the 20-70-year age range, with a mean age of 30.90 years. The control group included 10 subjects in the 20-70-year age range, with a mean age of 37.30 years (Figure 1).

### Distribution of Study Subjects Based on Sex

Among leukoplakia study subjects, 6 (60.0%) were male and 4 (40.0%) were female while among OSMF study subjects, 6 (60.0%) were male and 4 (40.0%) were female. In the control group subjects, 5 (50.0%) were male and 5 (50.0%) were female.

### Distribution Pattern of EGFR and Comparison of Immunohistochemical Expression

A total of 30 sections in which (n=10) cases of OL, (n=10) cases of OSMF and (n=10) cases of normal mucosa were examined and compared for the immunohistochemical expression and distribution pattern of EGFR.

**Figure 2.**
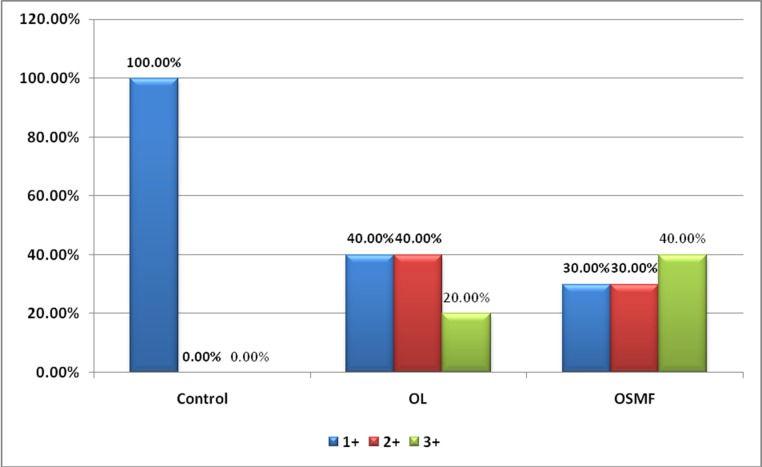
Photomicrographs showing immune reactions of epidermal growth factor receptor in: (A) normal mucosa showing positive expression in basal cells and a few suprabasal cells. (B) OL showing positive expression of membrane and cytoplasm of basal and suprabasal cells (×100). (C) OSMF showing positive expression in membrane and cytoplasm of basal and suprabasal cells and keratin layer (×40).

Histologically, all the proven dysplastic lesions of OL and OSMF showed varied intensities of staining with EGFR irrespective of the grade of dysplasia (Figure 2).

**Figure 3.**
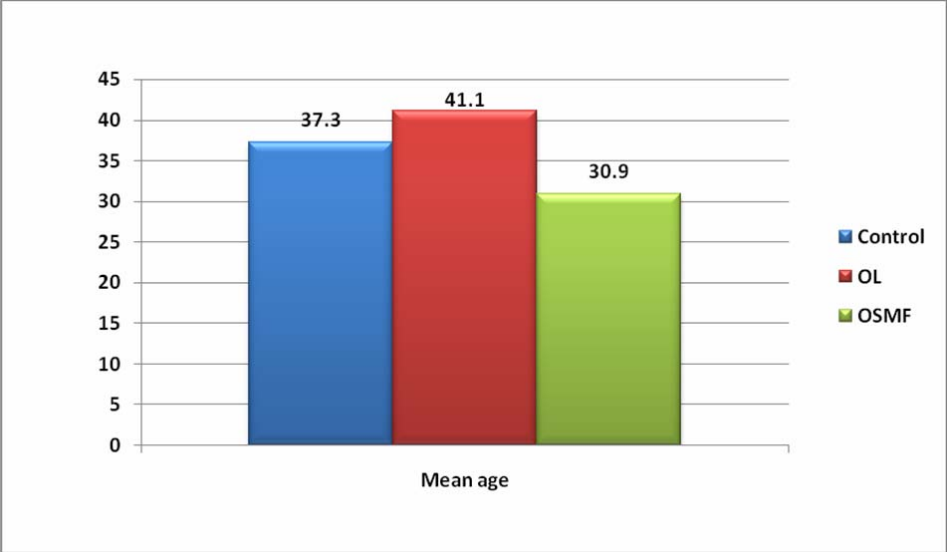
Comparison of the expression of EGFR in the normal oral mucosa, OL and OSMF. In the immunohistochemical expression of EGFR, of a total of 30 sections involving normal mucosa (control) (n= 10), OL (n=10) and OSMF (n=10). In the oral leukoplakia group (n=10), 4 cases (40%) showed weak expression with a score of 1+, 4 cases (40%) showed moderate expression with a score of 2+ and 2 cases (20%) showed strong expression with a score of 3+. In the oral submucous fibrosis group (n=10), 3 cases (30%) showed weak expression with a score of 1+, 3 cases (30%) showed moderate expression with a score of 2+ and 4 cases (40%) showed strong expression with a score of 3+.

In the oral leukoplakia group (n=10), 4 cases showed weak expression with a score of 1+, 4 cases showed moderate expression with a score of 2+ and 2 cases showed strong expression with a score of 3+. In the oral submucous fibrosis group (n=10), 3 cases showed a weak expression with a score of 1+, 3 cases showed moderate expression with a score of 2+ and 4 cases showed strong expression with a score of 3+. A statistical analysis was carried out using Pearson and chi-squared test to compare the EGFR expression quantitatively among the study and control groups and it was found to be statistically significant (P<0.05) (Table 1and Figure 3).

**Table 1:** Comparison of the expression of EGFR in normal oral mucosa, oral leukoplakia and oral submucous fibrosis

		**Score**						
		**1+**		**2+**		**3+**		**P-value**
		**N**	**%**	**N**	**%**	**N**	**%**	
	**Control**	10	100.0%	0	.0%	0	.0%
Group	**OL**	4	40.0%	4	40.0%	2	20.0%	0.012; Sig
	**OSMF**	3	30.0%	3	30.0%	4	40.0%	

## Discussion

The concept of a two-step process of cancer development in the oral mucosa, i.e., the initial presence of a precursor (pre-malignant, pre-cancerous) lesion subsequently developing into cancer, is well-established. At present, preference is given in the literature to the use of the adjective “potentially malignant” rather than to premalignant or precancerous. The presence of epithelial dysplasiais is generally accepted as one of the most important predictors of malignant development in PMDs. There is considerable uncertainty as to whether or not all clinically detectable lesions characterized as precursors will eventually develop into carcinoma. Many researches are trying to reduce these uncertainties that are aimed at diagnosing the malignant risk with the aid of molecular biomarkers that participate in carcinogenesis.

Oral carcinogenesis is a molecular and histological multistage process featuring genetic and phenotypic markers for each stage, which involves enhanced function of several tumor proto-oncogenes and/or the deactivation of tumor suppressor genes, resulting in the loss of cell cycle checkpoints, inhibition of normal apoptosis cycle and senescence. The progression towards malignancy includes sequential histopathological alterations ranging from hyperplasia through dysplasia to carcinoma in situ and invasive carcinoma, which are determined by the accumulation of a series of genetic and cellular events. In cellular carcinogenesis, various genes interact with each other, thus leading to multiple alterations that occur in a rather complex and integrated way and in different stages of progression of the disease.^10^ Several signal transduction pathways have been reported to be altered in cancer, leading to dramatic changes in cell survival, cell proliferation, morphology, angiogenesis, longevity and other properties, which characterize cancer cells.

Epidermal growth factor receptor (EGFR), one of the best studied biomarkers, plays an important role in the control of cellular proliferation, apoptosis, invasion, angiogenesis and metastasis as it works through the tyrosine kinase cascade. This receptor tyrosine kinase, also known as type I receptor tyrosine kinases or ErbB tyrosine kinase receptors, has many downstream signaling targets associated with carcinogenesis. EGF (epidermal growth factor) in oral epithelium exerts its biologic effect by binding to its receptor EGFR.^11^Once phosphorylated, the receptor can signal via the MAPK, Akt, ERK and Jak/STAT pathways. Abnormality of EGFR gene and over-expression of the protein have been reported in various human tumors, with an abnormal amplification of the EGFR gene being reported in oral squamous cell carcinoma, although not limited to the final stages of the carcinogenic process but also involved in the initiation and promotion of oral cancer. In fact, a low level of gene amplification upon binding to the membrane EGFR receptor also occurs at a significant frequency in epithelial dysplasia and carcinoma in situ, and, moreover, increased EGFR gene copies via amplification seems to play an important role in the development of invasive cancer.^12^ Moreover, the expression of proliferation markers TGF-α and EGFR in cells of the oral epithelium presenting a spectrum of dysplastic changes revealed a serial upregulation both in terms of area and intensity of staining of TGF-α in the epithelial cells of oral precancerous lesions exhibiting features of dysplasia. Likewise, TGF-α expression has been reported higher than EGFR’s in the proliferative pool of the oral epithelium in oral precancerous lesions, thus suggesting that an initial up-regulation of TGF-α was likely to exert a paracrine effect on the adjacent non-proliferative cells, therefore increasing the expression of the cell surface receptor.^5^ A significant linear increase in the intensity of staining of EGFR in the differentiated cells of the stratum spinosum in oral leukoplakia with mild epithelial dysplasia has also been reported, which might be explained by the inductive role of TGF-α over DNA synthesis in non-cycling cells.^5^

Researchers have reported that the over-expression of EGFR and other growth factors with similar structural and functional capacities is associated with several malignancies of breast, ovary, stomach, lung, colon and pancreas.^13-17^ EGFR over-expression has been correlated with poor prognosis in some human cancers and is apparently predictive of disease-free survival independent of cervical lymph node status. Studies in patients with breast cancer showing a link between clinical factors and over-expression of EGFR led to the successful treatment of chemotherapy-resistant breast tumors with agents that interfere with receptor tyrosine kinases. EGFR is over-expressed in embryonic oral tissues and in a variety of head and neck cancers.^18-21^However, their clinical significance in the head and neck tumors has hardly been investigated and remains unclear. Most of the studies reported were documented on the correlation of EGFR with oral squamous cell carcinoma while there are quite a few studies elaborating the association between oral potentially malignant lesions and the upregulation of EGFR receptor. Also over the past decade several studies have attempted to identify specific biomarkers to predict the malignant potential of more widely prevalent oral PMDs, OL.^8^ However, only few studies have addressed the molecular markers of malignant transformation in OSMF. Hence the present study was undertaken to study the quantitative immunoexpression of EGFR in the OL and OSMF in order to evaluate the malignant potential which was believed to be important in the early preventive and interventional approaches to the oral premalignant lesions before progressing to carcinoma.

The samples in which we noticed the presence of dysplasia were considered for staining with EGFR, but we did not consider the grade or severity of dysplasia. We tried to see the intensity of staining with EGFR in all the histologically proven dysplastic OL and OSMF. We correlated the presence of dysplasia with the positive expression of EGFR where all the cases that showed varied intensities of staining with EGFR were planned for prophylactic systemic administration of antioxidants, habit cessation, intralesional therapy for OSMF, surgical excision of OL which are also been regularly followed up to see the mucosal changes.

In the present study EGFR was expressed in all the samples of OL (n=10), OSMF (n=10) and normal mucosa (n=10). Our study showed the expression of EGFR in the cell membrane of squamous cells of basal and parabasal layers in normal mucosa. Similar findings have been reported by many authors: Srinivasan et al (2001),^5^Kobayashi et al (2013)^22^andRibeiro et al (2012).^23^Although normal mucosa of different sites were included, there was no correlation between the location of normal oral mucosa (NOM) and EGFR immunoexpression.

When leukoplakia slides were analyzed, expression of EGFR was not restricted to cell membrane of basal and parabasal layer but it was observed in various layers of the epithelium. However, our results differed from those reported by Ribeiro et al.^23^

In the present study, the findings in the expression intensity in cases of the oral submucous fibrosis group exhibited interesting considerations; for example, four (40%) out of ten cases showed a strong expression of EGFR and in the other group comprising oral leukoplakia two (20%) out of ten cases showed strong expression of EGFR; this variability may be due to the atrophy of the epithelium in OSMF, which is attributed to the increased loss of cells from the surface despite increased proliferative activity.

In the present study (n=10) in cases of leukoplakia, out of 10 cases 4 cases showed weaker expression and were scored as 1+, 4 cases showed moderate expression and were scored as 2+ and 2 cases showed strong expression and were scored as 3+. EGFR was expressed in leukoplakia regardless of grade of dysplasia, where positive staining was seen in all the cases of OL with histologically proven dysplasia, irrespective of its grading. Over-expression of EGFR in study cases suggested more aggressive behavior, which may be attributable to the activation of different signaling pathways that control diverse biological processes.

Oral submucous fibrosis is the most common premalignant condition in the oral cavity, affecting the native Asians.^24^ In our study, there was male predominance in the occurrence of the OSMF in the total sample. All the individuals affected with OSMF belonged to younger age group, consistent with other studies.^25^ Also the period of exposure at the time of initial reporting was shorter, which can be explained by the increased frequency of chewing, variations in the compositions of the packaged pan-masalas or ghutkas, and genetic predisposition of the individuals in the present cohort. This finding in our study supports the hypothesis that the relative risk of developing OSMF is directly proportional to the exposure to the harmful substances in a given period of time than the total duration of exposure.^26^

In the present study (n=10) out of 10 cases of OSMF, 3 cases showed weaker expression and were scored as 1+, 3 cases showed moderate expression and were scored as 2+ and 4 cases showed strong expression and were scored as 3+. The over-expression in study cases suggested that it may be due to the atrophy of epithelium, which has been attributed to the increased loss of cells from the surface, predisposing to the increased carcinogen exposure despite increases in proliferative activity.

The overall EGFR immunoexpression profile showed positive staining in all the samples of OL and OSMF with weaker expression in all the tissue samples of NOM with a P-value of 0.012 (significant). This suggests that this may be taken as an early marker of malignancy. A close follow-up of the dysplastic and clinically suspicious lesions is necessary in the routine clinical diagnostic workup. This indicates the upregulation of EGFR in the pathogenesis of oral carcinomas.

EGFR expression levels in the premalignant lesions appear to be a sensitive factor in predicting the neoplastic potential of dysplastic tissues. This suggests that EGFR may serve as a biological marker to identify high-risk subgroups and guide prophylactic therapy. The extensive research on molecular biomarkers of head and neck cancer influencing the cytogenetic alterations in the process of carcinogenesis has widened the scope of novel targeted therapies and chemopreventive trials. Given the role EGFR plays in the progression of tumors and its importance in early diagnosis of precancerous lesions, EGFR quickly became an interesting target for the development of novel therapies in the form of monoclonal antibodies to the extracellular domain of EGFR and small-molecule inhibitors of the intracellular tyrosine kinase domain. In the future it may be possible to improve the efficacy of antibodies by attaching bacterial toxins or radionuclides allowing specific delivery to tumor cells. The EGFR ligand, transforming growth factor (TGF)-α, has been linked to the *Pseudomonas* endotoxin TP-38 and is currently in phase I/II clinical trials (TGF-α-PE38 immunotoxin [TP-38]) employing intra-tumoral administration. It can be hypothesized from our study that all the PMDs showing positive EGFR expression should be considered under malignant risk and necessary intervention should be undertaken immediately along with regular follow-up.

## Conclusion

In conclusion, the results of this study suggested that EGFR over-expression can be one of the useful diagnostic markers for predicting the potential biologic behavior of OL and OSMF transforming into oral squamous cell carcinoma. Its over-expression gives a clue regarding the initiation or promotion of carcinogenesis. However, its over-expression in different oral leukoplakias and oral submucous fibrosis is yet to be revealed clearly in further studies.

All PMDs with dysplasia showing increased intensity of staining with EGFR, irrespective of the grade or severity of dysplasia, should be treated with surgery, supportive chemopreventive agents and habit cessation counseling until the mucosa shows no dysplasia in subsequent biopsy along with regular follow-up on semi-annual basis. Comprehension of the underlying pathways governing the progression of oral premalignant lesions is of the utmost importance. Owing to the targeted therapy against EGFR in many cancers in the body, clinical trials are underway inhibiting the EGFR and/or its downstream signal transduction pathways in the initial and intermediate stages of cancer development, thereby presenting a promising strategy for the development of chemotherapeutic and chemopreventive agents in oral squamous cell carcinoma. Hence, it is to be hoped that these biomarkers, including EGFR, could be used as intermediate end-points in the chemoprevention trials in near future.
